# Deep Neural Network-Based Method for Detecting Central Retinal Vein Occlusion Using Ultrawide-Field Fundus Ophthalmoscopy

**DOI:** 10.1155/2018/1875431

**Published:** 2018-11-01

**Authors:** Daisuke Nagasato, Hitoshi Tabuchi, Hideharu Ohsugi, Hiroki Masumoto, Hiroki Enno, Naofumi Ishitobi, Tomoaki Sonobe, Masahiro Kameoka, Masanori Niki, Ken Hayashi, Yoshinori Mitamura

**Affiliations:** ^1^Department of Ophthalmology, Tsukazaki Hospital, Himeji, Japan; ^2^Rist Inc., Tokyo, Japan; ^3^Department of Ophthalmology, Institute of Biomedical Sciences, Tokushima University Graduate School, Tokushima, Japan; ^4^Hayashi Eye Hospital, Fukuoka, Japan

## Abstract

The aim of this study is to assess the performance of two machine-learning technologies, namely, deep learning (DL) and support vector machine (SVM) algorithms, for detecting central retinal vein occlusion (CRVO) in ultrawide-field fundus images. Images from 125 CRVO patients (*n*=125 images) and 202 non-CRVO normal subjects (*n*=238 images) were included in this study. Training to construct the DL model using deep convolutional neural network algorithms was provided using ultrawide-field fundus images. The SVM uses scikit-learn library with a radial basis function kernel. The diagnostic abilities of DL and the SVM were compared by assessing their sensitivity, specificity, and area under the curve (AUC) of the receiver operating characteristic curve for CRVO. For diagnosing CRVO, the DL model had a sensitivity of 98.4% (95% confidence interval (CI), 94.3–99.8%) and a specificity of 97.9% (95% CI, 94.6–99.1%) with an AUC of 0.989 (95% CI, 0.980–0.999). In contrast, the SVM model had a sensitivity of 84.0% (95% CI, 76.3–89.3%) and a specificity of 87.5% (95% CI, 82.7–91.1%) with an AUC of 0.895 (95% CI, 0.859–0.931). Thus, the DL model outperformed the SVM model in all indices assessed (*P* < 0.001 for all). Our data suggest that a DL model derived using ultrawide-field fundus images could distinguish between normal and CRVO images with a high level of accuracy and that automatic CRVO detection in ultrawide-field fundus ophthalmoscopy is possible. This proposed DL-based model can also be used in ultrawide-field fundus ophthalmoscopy to accurately diagnose CRVO and improve medical care in remote locations where it is difficult for patients to attend an ophthalmic medical center.

## 1. Introduction

Central retinal vein occlusion (CRVO) is a vascular disease of the eye and a known cause of significant visual morbidity, including sudden blindness [1]. Pathogenesis of CRVO is believed to follow the principles of Virchow's triad of thrombogenesis, namely, vessel damage, stasis, and hypercoagulability [2]. In CRVO, the fundus may show retinal hemorrhages, dilated tortuous retinal veins, cotton-wool spots, optic edema, and macular edema (ME); ME is the most important cause of visual impairment in CRVO [3]. Intravitreous injections of antivascular endothelial growth factor (VEGF) agents have been shown to significantly improve visual acuity in eyes with CRVO-associated ME [4]. However, any delay in treatment with anti-VEGF agents results in poor functional improvement, and it is difficult to subsequently achieve satisfactory improvement in vision [5–7].

Thus, it is important to treat CRVO patients in an ophthalmic specialty center immediately after the onset to preserve visual function. However, establishing a large number of such centers is impractical because of rising public healthcare costs, a problem that is burdening several nations worldwide [8].

**Figure 1:**
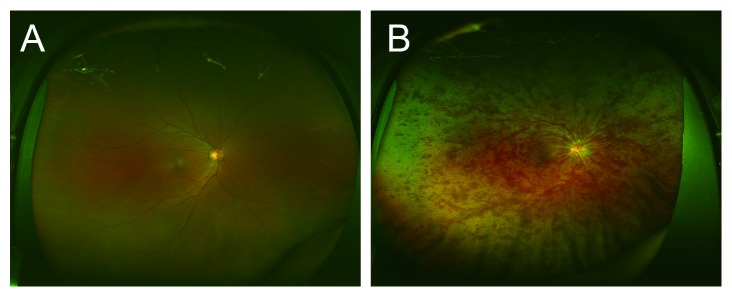
Representative fundus images obtained using ultrawide-field scanning laser ophthalmoscopy. Ultrawide-field fundus images of the right eye without central retinal vein occlusion (CRVO) (A) and with CRVO (B).

Recent remarkable advances in medical equipment include the ultrawide-field scanning laser ophthalmoscope, the Optos 200T× (Optos PLC, Dunfermline, United Kingdom). The Optos can easily and noninvasively provide wide-field fundus images (Figure 1) without mydriatic agent use, and it has been used for diagnosing or monitoring multiple conditions and for treatment evaluation in peripheral retinal and vascular pathology [9]. Importantly, if pupillary block and elevated intraocular pressure associated with dilation can be avoided, a trained nonmedical personnel can safely capture images to use them in telemedicine applications, especially in areas without ophthalmologists.

Image processing approaches using two machine-learning algorithms, namely, deep learning (DL) and support vector machines (SVMs), have retained investigator attention for years because of their extremely high-performance levels; in fact, increasing number of studies have assessed their applications in medical imaging [10–14]. Nonetheless, in ophthalmology, the use of image processing technology that uses DL algorithms and SVM models to analyze medical images has been previously reported [13, 15, 16]. However, to the best of our knowledge, no study has evaluated the possibility of automated CRVO diagnosis using Optos images and machine-learning technology. Therefore, in this study, we assessed the ability of a DL model to detect CRVO using Optos images and compared the results between DL- and SVM-based algorithms.

## 2. Materials and Methods

### 2.1. Image Dataset

Optos images of patients with acute CRVO and those without fundus diseases were extracted from the clinical database of the ophthalmology departments of the Tsukazaki Hospital, Tokushima University Hospital, and Hayashi Eye Hospital. These images were reviewed by a retinal specialist and stored in an analytical database. Of the 363 fundus images selected, 125 were from CRVO patients and 238 were from non-CRVO healthy subjects.

We used *K*-fold cross validation in this study, and it has been described in detail elsewhere [17, 18]. Briefly, image data were divided into *K* groups, and (*K* − 1) groups were used as training data, whereas one data group was used for validation. This process was repeated *K* times until each of the *K* groups became a validation dataset. The number of groups (*K*) was calculated using Sturges' formula (*K* = 1 + log_2_ *N*). Sturges' formula is used to decide the number of classes in the histogram [19, 20]. Thus, in this study, we categorized the data into nine groups.

Images in the training dataset were augmented by adjusting for brightness, gamma correction, histogram equalization, noise addition, and inversion so that the amount of training data increased by 18-fold. The deep convolutional neural network (DNN) model, as detailed below, was created and was trained using preprocessed image data.

This study was conducted in compliance with the principles of the Declaration of Helsinki and was approved by the ethics committees of Tsukazaki Hospital, Tokushima University Hospital, and Hayashi Eye Hospital.

### 2.2. Deep Learning Model and Training

**Figure 2:**
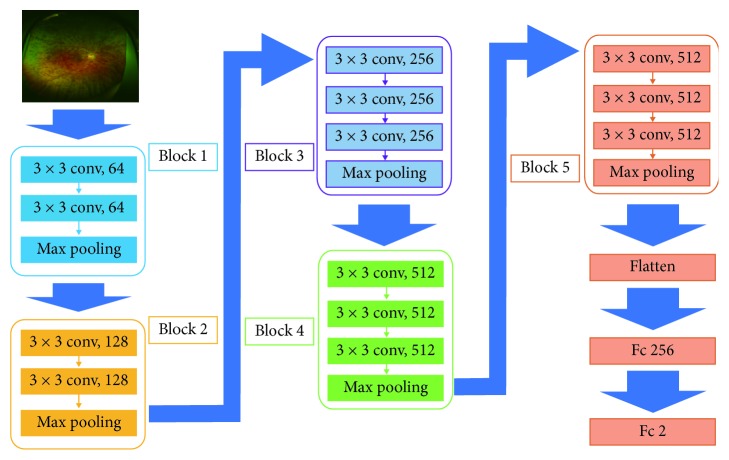
Overall architecture of Visual Geometry Group-16 model. Visual Geometry Group-16 (VGG-16) comprises five blocks and three fully connected layers. Each block includes convolutional layers followed by a max-pooling layer. Flattening of the output matrix after block 5 resulted in two fully connected layers for binary classification. The deep convolutional neural network used ImageNet parameters; the weights of blocks 1–4 were fixed, whereas the weights of block 5 and the fully connected layers were adjusted.

A DNN model called the Visual Geometry Group-16 (VGG-16) [21] was used in the present study, and its schematic is shown in Figure 2. This type of DNN is configured to automatically learn local features of images and generate a classification model [22–24]. The aspect ratio of the original Optos images was 3,900 × 3,072 pixels; however, for analysis, we changed the aspect ratio of all input images and resized them to 256 × 192 pixels. As the RGB input of images had a range of 0–255, it was first normalized to a range of 0–1 by dividing it by 255.

The VGG-16 model comprises five blocks and three fully connected layers. Each block includes convolutional layers followed by a max-pooling layer with decreasing position sensitivity but greater generic recognition [25]. Flattening of the output from block 5 results in only two fully connected layers. The first layer removes spatial information from the extracted feature vectors, and the second layer is a classification layer that uses feature vectors from target images acquired in previous layers in combination with the softmax function for binary classification. To improve generalization performance, dropout processing was performed such that masking was achieved with a probability of 25% in the first fully connected layer.

Fine tuning was used to increase the learning speed and achieve higher performance with lower quantitates of data [26, 27]. We used the following parameters from ImageNet: blocks 1 to 4 were fixed, whereas block 5 and the fully connected layers were trained.

The weights of block 5 and the fully connected layers were updated using the optimization momentum stochastic gradient descent algorithm (learning coefficient = 0.0005, inertial term = 0.9) [28, 29]. Of the 40 DL models obtained in 40 learning cycles, the one with the highest rate of correct answers for the test data was selected as the DL model to be evaluated in this study. For this purpose, Keras (https://keras.io/ja/) was run on TensorFlow (https://www.tensorflow.org/) written in Python and was used to build and evaluate the model. We trained the model using the CPU of Core (TM) i7-8700K by Intel and the GPU of GeForce GTX 1080 Ti by NVIDIA.

### 2.3. Support Vector Machine Model

We used the soft-margin SVM implemented in the scikit-learn library using the radial basis function kernel [30]. We reduced all images to 60 dimensions as this was the number of dimensions that was found to provide the highest correct answer rate for the test data; for this, we tested 10–70 dimensions in steps of 10. The optimal values for cost parameter “*C*” of the SVM algorithm and parameter “*γ*” of the radial basis function were determined by grid search using quadrant cross validation, and the combination with the highest average correct answer rate was selected. The parameter values tested for *C* were 1, 10, 100, and 1000 and those for *γ* were 0.0001, 0.001, 0.01, 0.1, and 1. The final learning model was generated using the optimized parameter values of *C* = 10 and *γ* = 0.0001.

### 2.4. Outcomes

Receiver operating characteristic (ROC) curves for CRVO were created on the basis of the ability of the DL and SVM models to distinguish between CRVO and non-CRVO images, and the models were compared using area under the curve (AUC), sensitivity, and specificity values.

### 2.5. Heat Map

A heat map of the DNN focus site was created and classified using gradient-weighted class activation mapping [21]. Next, composite images were created by overlaying heat maps of the DNN focus site on the corresponding CRVO and non-CRVO images. The third convolution layer in block 3 was defined as the target layer, and the rectified linear unit was used as the backprop modifier. This process was performed using Python Keras-vis (https://raghakot.github.io/keras-vis/).

### 2.6. Statistical Analysis

Patient demographic data such as age were compared using Student's *t*-test, whereas Fisher's exact test was used for comparing the gender ratio and the ratio of the right to left eye images.

The 95% confidence interval (CI) of AUC was obtained as follows. Images judged to exceed a threshold were defined as positive for CRVO, and the ROC curve was created. We created nine such models and nine ROC curves. For determining AUC, the 95% CI was obtained by assuming normal distribution and using the average and standard deviation of the nine ROC curves. For estimating sensitivity and specificity, optimal cutoff values, which are the points closest to the point at which both sensitivity and specificity are 100% in each ROC curve, were used [26]. The sensitivity and specificity at the optimal cutoff value were calculated using the Youden index [31]. The ROC curve was calculated using scikit-learn, and CIs for sensitivity and specificity were determined using SciPy. The paired *t*-test was used to compare AUCs between the DL and the SVM models.

## 3. Results

We used 125 CRVO images from 125 patients (mean age, 67.8 ± 13.9 years; 67 men and 58 women; 61 left fundus and 64 right fundus images) and 238 non-CRVO images from 202 subjects (mean age, 68.6 ± 7.9 years; 104 men and 98 women; 122 left fundus and 116 right fundus images) in this analysis. No significant differences were detected between these two groups with respect to age, gender ratio, and left-right eye image ratio (Table 1).

**Figure 3:**
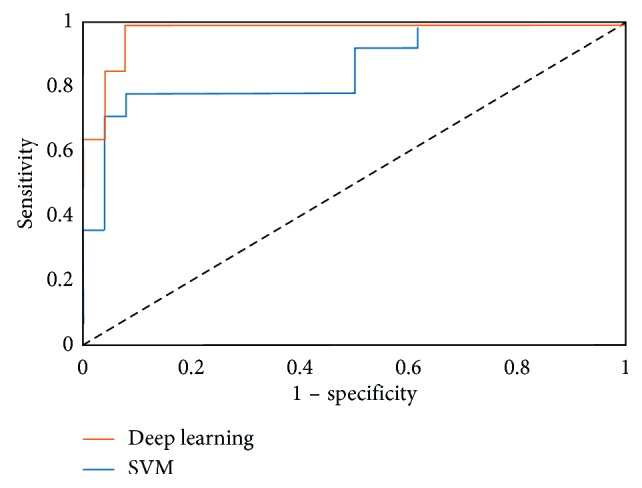
Receiver operating characteristic (ROC) curve for central retinal vein occlusion.

The DL model's sensitivity for diagnosing CRVO was 98.4% (95% CI, 94.3–99.8%), its specificity was 97.9% (95% CI, 94.6–99.1%), and the AUC was 0.989 (95% CI, 0.980–0.999); in contrast, sensitivity of the SVM model was 84.0% (95% CI, 76.3–89.3%), its specificity was 87.5% (95% CI, 82.7–91.1%), and the AUC was 0.895 (95% CI, 0.859–0.931). In ROC curves, AUC of the DL model was significantly higher than that of the SVM model (*P* < 0.001) (Figure 3).

**Figure 4:**
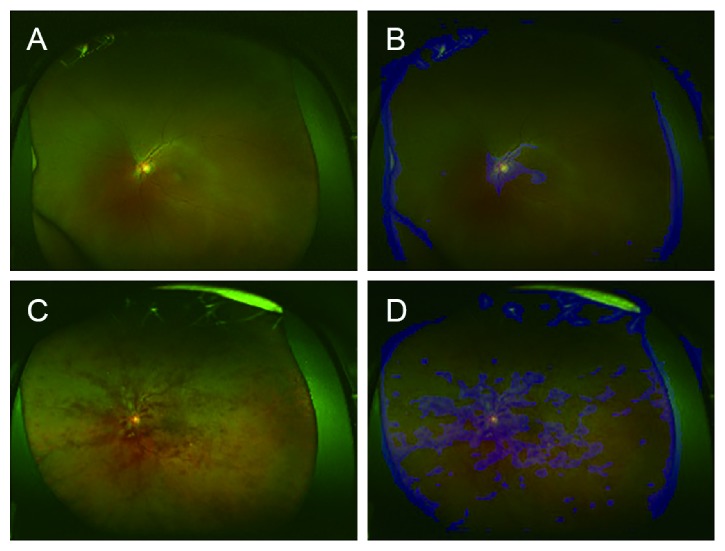
Representative ultrawide-field fundus images and corresponding heat maps. The ultrawide-field fundus image without central retinal vein occlusion (CRVO) (A), and its corresponding superimposed heat map (B); with CRVO (C), and its corresponding superimposed heat map (D). In the image without CRVO (A), the deep convolution neural network focused on the optic disc (blue), whereas in the image with CRVO (B), the model focused on the optic disc and on the retinal hemorrhages (blue) (D).

A composite image, comprising the fundal image superimposed with its corresponding heat map, was created by the DNN, and these images showed that DNNs could accurately identify crucial areas in the fundal images; a representative composite image is presented in Figure 4. Blue was used to indicate the strength of DNN-based identification, and an increase in color intensity was observed in areas with retinal hemorrhage and at the focus points. Thus, in non-CRVO images, the heat map showed that focal points accumulated around the optic disc, whereas in CRVO images, focal points accumulated around the optic disc and around retinal hemorrhages. These results imply that DNNs may be able to distinguish between CRVO eyes and normal eyes by identifying and highlighting retinal hemorrhages.

## 4. Discussion

The fundamental aim of this study was to explore the possibility of early detection of CRVO from Optos fundus photographs using DL-based algorithms. If screening for CRVO is possible noninvasively and without the use of mydriatic agents, this approach would be medically viable. Currently, it is unreasonable to expect ophthalmologists to interpret all Optos-acquired fundus images because of associated medical resource costs. Therefore, a DL model that can accurately diagnose conditions based on ultrawide-field fundus ophthalmoscopy images without the need for human input can be used to screen and diagnose a very large number of patients at a very low cost.

Here, we have used DL technology to identify Optos images that show presence of CRVO. Our results show that the DL model has higher sensitivity, specificity, and AUC values than the SVM model for detecting CRVO in Optos-derived fundus photographs.

Further, using heat maps, we show that DNN could accurately identify an area around the optic disc in the non-CRVO images, whereas in CRVO images, it focused on the area around the optic disc and could highlight retinal hemorrhages. This result implies that the proposed DNN model may be able to identify CRVO by focusing on areas with suspected retinal hemorrhages. It is known that DL algorithm-based models can automatically learn local feature values of images and generate classification models [22, 26, 29, 31]. Additionally, DL includes several layers for the identification of local features of complicated differences, which can subsequently be combined [29].

In recent years, a number of studies have addressed that CNN hugely outperforms classic ML algorithms in image classification tasks [16, 32–34]. Recently, Wang et al. have reported that the performance of the DL model was not significantly different from that of the best classical methods, including SVM and human physicians, when classifying mediastinal lymph node metastasis in nonsmall cell lung cancer using positron emission tomography/computed tomography images [35]. This could be because image information necessary for classification was lost during image convolution in DL. In contrast, we found that the performance of the DL model was better than that of the SVM model in accurately diagnosing CRVO using Optos images. As most cases of CRVO need early intervention, patients diagnosed with CRVO using this method can immediately consult retinal specialists and receive necessary advanced treatment at an ophthalmic medical center. The Optos-based telemedicine technology being proposed here could significantly help us in preserving good visual function in CRVO patients living in areas with inadequate ophthalmic care and could potentially be used to cover large areas without adequate care facilities.

Despite the above, our study has a few limitations. First, we have only compared images of normal retinas with CRVO retinas and did not include images of other retinal diseases. Based on the image examples presented in this study, it may be expected that a CNN algorithm should easily be able to classify between the two types of images. To use this model under clinical conditions, further development and testing to ensure accurate identification of multiple conditions other than CRVO would be essential. Additionally, clarity of the eye may decrease in patients with mature cataract or severe vitreous hemorrhage, and in such cases, analysis of images captured using Optos may be difficult. Thus, future studies should extensively evaluate the performance and versatility of DL using larger samples and with images of other fundus diseases.

## 5. Conclusions

In conclusion, the DL model performed better than the SVM model in terms of its ability to distinguish between CRVO and normal eyes using ultrawide-field fundus ophthalmoscopic images. This technology has significant potential clinical usefulness as it can be combined with telemedicine to reach large areas where no specialist care is available.

## Figures and Tables

**Table 1:** Patient demographics.

	CRVO	Non-CRVO	*p* value
Number of images (patients)	125 (125)	238 (202)	—
Age (yrs)	67.8 ± 13.9	68.6 ± 7.9	0.489 (Student's *t*-test)
Sex, female	58 (46.4%)	98 (48.5%)	0.734 (Fisher's exact test)
Left fundus	61 (48.8%)	122 (51.3%)	0.660 (Fisher's exact test)

## Data Availability

The Optos image datasets and its corresponding superimposed heat maps analyzed during the current study are available from the corresponding author on reasonable request.
